# Circulating 20S Proteasome Is Independently Associated with Abdominal Muscle Mass in Hemodialysis Patients

**DOI:** 10.1371/journal.pone.0121352

**Published:** 2015-03-24

**Authors:** Hirotaka Fukasawa, Mai Kaneko, Hiroki Niwa, Takashi Matsuyama, Hideo Yasuda, Hiromichi Kumagai, Ryuichi Furuya

**Affiliations:** 1 Renal Division, Department of Internal Medicine, Iwata City Hospital, Iwata, Shizuoka, Japan; 2 First Department of Medicine, Hamamatsu University School of Medicine, Hamamatsu, Shizuoka, Japan; 3 The Department of Clinical Nutrition, School of Food and Nutritional Sciences, University of Shizuoka, Shizuoka, Shizuoka, Japan; University of Louisville School of Medicine, UNITED STATES

## Abstract

Protein-energy wasting is highly prevalent in hemodialysis patients, and it contributes to patient morbidity and mortality. The ubiquitin-proteasome system is the major pathway for intracellular protein degradation and it is involved in the regulation of basic cellular processes. However, the role of this system in the determination of nutritional status is largely unknown. To examine a relationship between protein-energy wasting and the ubiquitin-proteasome system, a cross-sectional study of 76 hemodialysis patients was performed. Plasma concentrations of 20S proteasome were studied to evaluate its association with muscle and fat mass, which were investigated by abdominal muscle and fat areas measured using computed tomography and by creatinine production estimated using the creatinine kinetic model. Plasma 20S proteasome concentrations significantly and negatively correlated with abdominal muscle areas and creatinine production (*rho* = -0.263, *P* < 0.05 and *rho* = -0.241, *P* < 0.05, respectively), but not abdominal subcutaneous and visceral fat areas. Multiple regression analyses showed that 20S proteasome was a significant independent predictor of abdominal muscle area (*P* < 0.05). In conclusion, plasma 20S proteasome concentrations were independently associated with abdominal muscle mass in hemodialysis patients. Our findings indicate a relationship between circulating 20S proteasomes and muscle metabolism in these patients.

**Trial Registration:**

UMIN Clinical Trials Registry UMIN000012341

## Introduction

Protein-energy wasting (PEW) is a condition associated with chronic kidney disease (CKD), and it is characterized as decreased body stores of muscle and fat mass [1]. PEW is present in a large proportion of advanced CKD patients, and it is related to increased morbidity and mortality [2]. Inadequate nutrition, inflammation, perturbations of appetite-controlling hormones, insulin resistance, and metabolic acidosis may contribute to the pathogenesis of PEW [3, 4].

Protein degradation via the ubiquitin-proteasome system (UPS) is the major pathway of the non-lysosomal proteolysis, and this system plays important roles in a variety of fundamental cellular processes, such as the regulation of cell cycle progression, apoptosis, sodium channel function, fibrosis, and the modulation of inflammatory responses [5, 6]. UPS also plays a critical role in muscle atrophy during various catabolic states, including sepsis, burn injuries, cancer, diabetes and uremia [7]. The 20S proteasome is a central element of intracellular UPS [8].

Recently, a growing body of evidence demonstrated the presence of 20S proteasome both intracellularly and in the extracellular space, where it may exert physiological functions [9]. Furthermore, 20S proteasomes are physiologically present in the human circulation, and increased concentrations were demonstrated in diverse pathological states, such as autoimmune diseases [10], sepsis, acute respiratory distress syndrome [11] and several cancers [12–14]. However, the role of circulating 20S proteasomes has not been investigated in patients with CKD.

This study reports cross-sectional data from a well-characterized cohort of patients undergoing maintenance HD. We also assessed associations between circulating 20S proteasomes and several nutritional markers in these patients.

## Materials and Methods

### Subjects

Seventy-six patients (50 men, 26 women) who had been undergoing HD for at least three consecutive months at Iwata City Hospital (Shizuoka, Japan) were enrolled in this cross-sectional study. All patients were subjected to regular HD for 4–5 hours three times per week at a blood flow rate of 180–240 mL/min. All patients used bicarbonate dialysate (Kindaly AF-2E, Fuso, Osaka, Japan) at a dialysate flow rate of 500 mL/min.

The institutional ethics committee approved the study protocol, and all patients provided informed consent before participation. This study was also registered with the Clinical Trial Registry of the University Hospital Medical Information Network (http://www.umin.ac.jp/, study number: UMIN000012341).

### Anthropometric measurements

Body weight was measured before and after each dialysis session, and the post-dialysis body weight of each patient was used as his or her dry weight (DW). Body mass index (BMI, kg/m^2^) was calculated by dividing the DW (kg) by the squared height (m).

### Blood sampling and laboratory examinations

Blood samples of patients were drawn at the beginning and end of the first dialysis session of the week, following a 2-day interval. As a control, blood samples from 5 healthy adults were also drawn. Plasma samples were separated immediately and stored at -80°C until analyzed. Serum electrolytes, urea nitrogen, creatinine (Cr), albumin, cholesterol, triglyceride, and C-reactive protein (CRP) levels were measured using standard laboratory techniques with an auto-analyzer. Plasma 20S proteasome and interleukin-6 (IL-6) concentrations were measured using enzyme-linked immunosorbent assays (ELISA, Proteasome ELISA kit: Enzo Life Sciences, Farmingdale, NY, USA and Ultra-sensitive human IL-6 immunoassay kit: R&D Systems, Minneapolis, MN, USA, respectively).

### Evaluation of 20S proteasome concentrations using Western blot analysis

Equal amounts of plasma samples (0.8 μL) after dilution were loaded for sodium dodecyl sulfate-polyacrylamide gel electrophoresis (SDS-PAGE) as described previously [15]. The primary antibody was a mouse monoclonal anti-20S proteasome (alpha6 subunit, clone MCP20, Enzo Life Sciences, Farmingdale, NY, USA), whish was also used as the capture antibody in ELISA described above. A 20S Proteasome Stock Solution (0.05 μg/mL, Enzo Life Sciences) was used as a standard. Band intensities were quantified using NIH-IMAGE, which is a public domain planimetry program available from the National Institutes of Health (written by Wayne Rasband, The National Institutes of Health, Bethesda, MD, USA), and compared with a standard of 20S proteasomes.

### Measurements of abdominal muscle and fat areas using computed tomography

Abdominal computed tomography (CT) scans were performed during a patient’s periodic check-up. The CT scan and measurement of Cr production were performed within 3 months of each other. Each patient was examined in the supine position and the thickness of each slice was 10 mm. Axial CT images for muscle and fat mass evaluations were obtained at the level of the third lumber spine. Radiographic images were digitally scanned for analyses on a personal computer. The adipose-tissue-free abdominal muscle area (AMA), abdominal subcutaneous fat area (ASFA) and abdominal visceral fat area (AVFA) were measured using NIH-IMAGE [16].

### Normalized protein equivalent of nitrogen appearance and evaluation of hemodialysis dose

The normalized protein equivalent of nitrogen appearance (nPNA) was calculated using the formula published by the K/DOQI Hemodialysis Adequacy Work Group [17]. Data collected during a beginning-of-the-week dialysis session were used for these calculations. HD dose was evaluated using the following formula:

$$Kt/{V\;}_{urea}\;=\;-\ln\left(R\;-0.008\;\times \;t\right)\;+\;\left[4\;–\left(3.5\;\times \;R\right)\;\times \;UF/W\right]$$

where Kt/V _urea_ is single-pool Kt/V _urea_, R is the ratio of post-dialysis to pre-dialysis serum urea nitrogen, t is time of dialysis in hours, UF is the amount of ultrafiltration in liters, and W is post-dialysis body weight in kilograms. nPNA was evaluated using the following formula:

$$nPNA\;\left(g/kg/day\right)\;=\;C_{0}/\left[36.3\;+5.48\;\left(Kt/{V\;}_{urea}\right)\;+\;53.5/\left(Kt/{V\;}_{urea}\right)\right]\;+0.168$$

where C_0_ is the pre-dialysis concentration of serum urea nitrogen in mg per deciliter.

### Geriatric nutritional risk index

The geriatric nutritional risk index (GNRI) was calculated from the patient’s serum albumin and body weight by using the equation developed by Bouillanne *et al*. [18] as follows:

$$GNRI\;=\;\left[14.89\;\times \;albumin\;\left(g/dl\right)\right]\;+\;\left[41.7\;\times \;body\;weight/ideal\;body\;weight\right]$$

### Estimation of creatinine production using the creatinine kinetic model

The Cr production rate was estimated using the pre- and post-dialysis Cr concentrations at the first dialysis session of the week based on the Cr kinetic model developed by Shinzato *et al*. [19] with slight modification, according to the following equation:

$$The\;Cr\;production\;rate\;\left(g/day\right)\;=\;C_{S}\left[7056/A\;+\;\Delta BW/IBW\;\times \;240/\left(72\;-\;T_{d}\right)\right]\;\times \;DW$$

where

$$A\;=\;3864\;+\;\left(7.8T_{d}\;+411\right)\;\ln\left(C_{e}/C_{s}\right)\;-1.5T_{d}-\;1,449/\left[\left(0.0190T_{d}+\;0.999\right)\;\ln\left(C_{e}/C_{s}\right)–\left(0.0036{7T}_{d}-0.0219\right)\right]$$

where C_s_ (mg/dL) is the pre-dialysis Cr concentration, C_e_ (mg/dL) is the post-dialysis Cr concentration, ΔBW (kg) is the body weight decrease resulting from dialysis, IBW (kg) is the ideal body weight and T_d_ (hour) is the dialysis duration.

### Statistical analysis

Data are expressed as the means ± standard deviation (SD) for continuous variables with normal distributions or the median and interquartile range (25th to 75th percentiles) for data with skewed distributions. The threshold for statistical significance was set at *P* < 0.05. Comparisons between two groups were performed using the Mann-Whitney *U*-test. Spearman’s rank-order correlation analysis was used to evaluate potential associations between the 20S proteasome and the selected parameters. Multivariate regression analyses assessed the independent predictors of AMA and AMA standardized for height. All statistical analyses were performed using StatView 5 (SAS Institute Inc., Cary, NC, USA) or IBM SPSS statistical software, version 19.0 (IBM SPSS, Tokyo, Japan).

## Results

### Causes of end-stage kidney diseases

The causes of end-stage kidney diseases in this study population were primary kidney diseases, such as chronic glomerulonephritis and nephrosclerosis in 63 patients (82%), overt diabetic nephropathy in 8 patients (11%) and polycystic kidney disease in 5 patients (7%).

### Plasma levels of 20S proteasome

**Fig 1:**
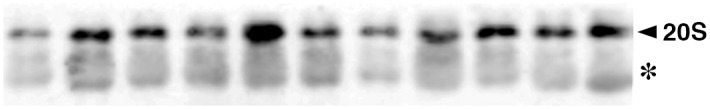
Western blot analysis of 20S proteasome in 11 patients. Bands of the 20S proteasome are detected (arrow head). Laddered bands were also present under the specific bands (asterisk).

Plasma levels of 20S proteasome measured with ELISA and Western blot analysis were significantly correlated each other (*P* < 0.05), but the ELISA values were slightly higher than Western blot analysis (1.34 ± 1.12 μg/mL and 1.33 ± 0.53 μg/mL, respectively, Table 1). Two patients demonstrated extremely high 20S proteasome levels when plasma samples were measured with ELISA (110.90 μg/mL and 70.10 μg/mL). Furthermore, the laddered bands, which might be included as levels when the samples were measured with ELISA, were present under the specific bands of 20S proteasome (Fig. 1). Therefore, we used the data measured with Western blot analysis in subsequent analyses.

**Table 1:** Patient Characteristics.

Variables	Total (n = 76)	Male (n = 50)	Female (n = 26)	*P*
Age, years	67.0 (60.0 to 73.3)	67.5 (60.3 to 74.0)	65.5 (59.8 to 71.5)	0.493
Dialysis vintage, months	142.5 (42.3 to 269.8)	136.0 (39.3 to 267.0)	157.5 (85.8 to 282.5)	0.536
Height, m	1.60 ± 0.09	1.64 ± 0.06	1.52 ± 0.08	<0.001
Dry weight, kg	50.3 ± 9.7	53.8 ± 8.9	43.5 ± 7.4	<0.001
BMI, kg/m^2^	20.3 ± 2.9	20.7 ± 2.8	19.6± 3.0	0.057
Systolic blood pressure, mmHg	157.3 ± 19.3	156.2 ± 22.1	159.5 ± 12.6	0.243
Diastolic blood pressure, mmHg	85.2± 13.0	82.8± 13.1	89.8 ± 11.8	<0.05
Hemoglobin, g/dL	11.4 ± 1.1	11.4 ± 1.1	11.4 ± 1.3	0.665
Total protein, g/dL	6.4 ± 0.4	6.4 ± 0.5	6.4 ± 0.4	0.965
Serum albumin, g/dL	3.7 ± 0.4	3.7 ± 0.4	3.7 ± 0.4	0.252
Total cholesterol, mg/dL	152.4 ± 32.5	145.1 ± 31.0	166.3 ± 31.1	<0.05
LDL choresterol, mg/dL	86.0 ± 20.8	83.4 ± 21.2	90.8 ± 19.6	0.114
Blood urea nitrogen, mg/dL	62.1 ± 12.7	64.2 ± 10.8	58.2 ± 15.1	<0.05
Serum creatinine, mg/dL	10.7 ± 2.7	11.1 ± 3.0	9.8 ± 1.7	<0.05
Sodium, mEq/L	139.3 ± 2.8	138.7 ± 2.7	140.4 ± 2.7	<0.05
Potassium, mEq/L	4.6 ± 0.5	4.7 ± 0.5	4.5 ± 0.6	0.277
Calcium, mg/dL	9.5 ± 0.9	9.3 ± 0.9	9.7 ± 0.8	0.116
Phosphate, mg/dL	5.0 ± 1.3	5.1 ± 1.4	4.8 ± 1.2	0.437
Intact PTH, pg/mL	101.3 ± 107.1	88.0 ± 53.4	127.0 ± 166.6	0.978
Beta_2_-microglobulin, mg/L	26.5 ± 6.6	26.1 ± 6.6	27.3 ± 6.7	0.669
Kt/V _urea_	1.6 ± 0.3	1.5 ± 0.2	1.9 ± 0.3	<0.001
nPNA, g/kg/day	0.96± 0.17	0.97± 0.15	0.94 ± 0.21	0.266
GNRI	91.8± 7.7	92.1 ± 7.7	91.2 ± 7.8	0.547
CRP, mg/dL	0.1 (0.0 to 0.3)	0.1 (0.1 to 0.4)	0.0 (0.0 to 0.1)	<0.01
IL-6, pg/mL	5.10 (3.10 to 10.45)	5.12(3.51 to 10.25)	4.65 (2.75 to 10.17)	0.529
20S Proteasome, ELISA	1.34 ± 1.12	1.26 ± 1.11	1.51 ± 1.13	0.233
20S Proteasome, Western blot	1.33 ± 0.53	1.28 ± 0.47	1.46 ± 0.66	0.244
AMA, cm^2^	96.5 (81.4 to 119.0)	114.6 (90.0 to 128.0)	78.7 (69.0 to 91.3)	<0.001
AMA standardized for height	61.1 (50.9 to 73.4)	68.9 (56.4 to 76.8)	51.6 (43.7 to 59.2)	<0.001
ASFA, cm^2^	70.6 (49.0 to 108.6)	67.2(46.1 to 106.0)	75.1 (58.8 to 121.6)	0.178
ASFA standardized for height	45.1 (31.9 to 64.7)	42.6 (27.9 to 62.3)	50.2 (36.8 to 77.9)	0.090
AVFA, cm^2^	47.8(22.1 to 87.2)	54.1 (22.7 to 110.8)	34.5 (21.9 to 58.8)	0.090
AVFA standardized for height	31.2 (14.0 to 54.0)	34.5 (13.8 to 69.8)	23.3 (14.1 to 38.1)	0.168
Creatinine production rate, g/day	0.83 (0.69 to 1.08)	0.99 (0.72 to 1.19)	0.74 (0.61 to 0.86)	<0.01

All variables are expressed as the means ± SD or the medians and interquartile range (25th to 75th percentiles).

Abbreviations: AMA, abdominal muscle area; ASFA, abdominal subcutaneous fat area; AVFA, abdominal visceral fat area; BMI, body mass index; CRP, C-reactive protein; GNRI, geriatric nutritional risk index; IL-6, interleukin-6; Kt/V _urea_, amount of dialysis delivered to each patient per treatment; LDL, low-density lipoprotein; nPNA, normalized protein equivalent of nitrogen appearance; PTH, parathyroid hormone.

### A comparison of 20S proteasome levels between patients and healthy subjects

In patients undergoing hemodialysis, plasma levels of 20S proteasome were relatively higher than those in healthy controls (1.33 ± 0.53 μg/mL vs. 0.95 ± 0.13 μg/mL, respectively, *P* = 0.110).

### Clinical profiles

Table 1 presents the characteristics of the study population. The median age was 67.0 years (the 25th to 75th percentile ranged from 60.0 to 73.3 years). The median dialysis vintage was 142.5 months (range, 42.3 to 269.8 months), and the mean BMI was 20.3 ± 2.9 kg/m^2^.

No significant sex differences were observed with respect to age, dialysis vintage, BMI, or serum albumin levels of the study participants. On the other hand, the patient height, dry weight (DW), serum Cr levels, AMA, AMA standardized for height and Cr production rate were significantly greater in men than women. No significant differences in IL-6 and 20S proteasome levels were observed between men and women.

### Correlations between 20S proteasome and clinical parameters

**Fig 2:**
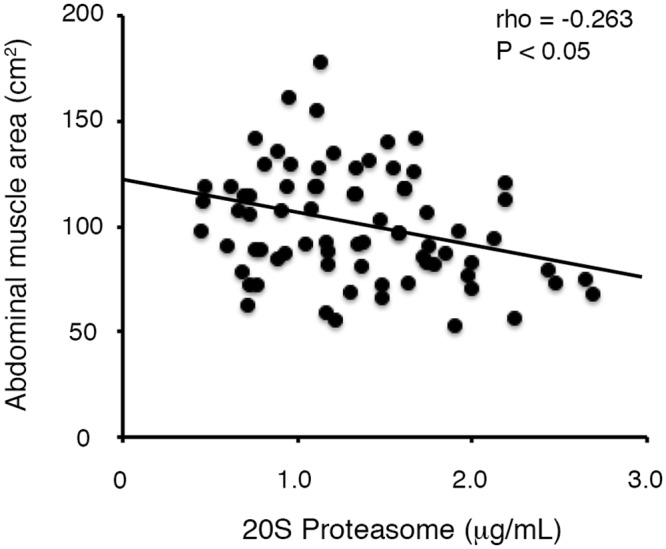
Association between abdominal muscle areas and 20S proteasome levels.

Significant negative correlations were observed between the 20S proteasome levels and dry weight (*P* < 0.05), phosphate (*P* < 0.001), intact PTH (*P* < 0.05), AMA (*P* < 0.05, Fig. 2), AMA standardized for height (*P* < 0.05) and the Cr production rate (*P* < 0.05). Conversely, the 20S proteasome levels were not correlated with age, gender, dialysis vintage, height, nPNA, GNRI, log-transformed IL-6, ASFA, ASFA standardized for height, AVFA and AVFA standardized for height (Table 2).

**Table 2:** Correlations between 20S Proteasome Concentrations and Clinical Variables.

	20S Proteasome	
	Correlation coefficient	*P*
Age	0.133	0.252
Gender, male	-0.135	0.246
Dialysis vintage	−0.146	0.207
Height	−0.154	0.183
Dry weight	−0.242	<0.05
BMI	−0.190	0.101
Systolic blood pressure	0.157	0.177
Diastolic blood pressure	−0.046	0.694
Total protein	0.072	0.535
Serum albumin	0.077	0.511
Total cholesterol	0.240	<0.05
LDL cholesterol	0.144	0.214
Blood urea nitrogen	−0.004	0.971
Serum creatinine	−0.185	0.110
Calcium	0.019	0.873
Phosphate	−0.391	<0.001
Intact PTH	−0.227	<0.05
Beta _2_-microglobulin	0.024	0.835
Kt/V _urea_	0.202	0.080
nPNA	0.049	0.675
GNRI	−0.107	0.358
Log IL-6	−0.144	0.216
AMA	−0.263	<0.05
AMA standardized for height	−0.266	<0.05
ASFA	−0.034	0.771
ASFA standardized for height	−0.018	0.874
AVFA	−0.038	0.745
AVFA standardized for height	−0.034	0.768
Creatinine production rate	−0.241	<0.05

Abbreviations: AMA, abdominal muscle area; ASFA, abdominal subcutaneous fat area; AVFA, abdominal visceral fat area; BMI, body mass index; GNRI, geriatric nutritional risk index; IL-6, interleukin-6; Kt/V _urea_, amount of dialysis delivered to each patient per treatment; LDL, low-density lipoprotein; nPNA, normalized protein equivalent of nitrogen appearance; PTH, parathyroid hormone.

### Determinants of abdominal muscle mass

Multiple regression analyses revealed that 20S proteasome was independently associated with AMA when 20S proteasome, age, gender, dialysis vintage, height, log-transformed IL-6 (model 1) and nPNA (model 2) were adjusted as independent variables (Table 3). The analyses also revealed that 20S proteasome was independently associated with AMA standardized for height when 20S proteasome, age, gender, dialysis vintage, log-transformed IL-6 and nPNA were adjusted as independent variables (*P* = 0.036).

**Table 3:** Multiple Regression Analysis using Abdominal Muscle Area (AMA) as the Dependent Variable and 20S Proteasome, Age, Sex, Dialysis Vintage, Height, Log IL-6 and nPNA as Independent Variables.

	Model 1		Model 2	
	*Beta*	*P*	*Beta*	*P*
Dependent variable: Abdominal muscle area (AMA)				
20S Proteasome	−0.190	0.037	−0.185	0.043
Age	−0.224	0.058	−0.229	0.054
Gender, male	0.464	0.001	0.468	0.001
Dialysis vintage	−0.094	0.274	−0.077	0.379
Height	0.155	0.289	0.163	0.266
Log IL-6	−0.135	0.140	−0.136	0.138
nPNA	-	-	−0.083	0.342

Abbreviations: IL-6, interleukin-6; nPNA, normalized protein equivalent of nitrogen appearance.

Furthermore, plasma 20S proteasome levels significantly and inversely decreased when the levels were divided into 2 groups according to AMA (1.49 ± 0.58 μg/mL in lower AMA group and 1.18 ± 0.54 μg/mL in higher AMA group, respectively, *P* < 0.05) and the Cr production rate (1.46 ± 0.56 μg/mL in lower Cr production rate group and 1.22 ± 0.51 μg/mL in higher Cr production rate group, respectively, *P* < 0.05).

## Discussion

The primary finding of this study is that plasma 20S proteasome levels are associated with abdominal muscle mass in HD patients, even after adjusting for potential confounding variables. In addition, 20S proteasome levels are significantly and negatively correlated with dry weight, serum phosphate levels and the Cr production rate. To our knowledge, this report is the first study to demonstrate a link between circulating 20S proteasome levels and muscle metabolism of patients with advanced CKD.

CKD patients often suffer from nutritional problems that are associated with increased morbidity and mortality [20]. PEW is a proposed term to describe the state of decreased body stores of protein and energy that occurs in CKD and is diagnosed if three characteristics are present in four categories: (1) serum chemistry (low serum levels of albumin, prealbumin, or cholesterol), (2) reduced body mass (low BMI, weight loss, or low body fat percentage), (3) reduced muscle mass (muscle wasting, reduced mid-arm muscle circumference, or creatinine appearance), and (4) reduced dietary intake (low intake of protein or energy) [1]. In fact, HD patients exhibit lower BMIs than age- and sex-matched control subjects from the general population [21] and a previous study showed that increased BMI contributed to survival advantages in dialysis patients [22]. Furthermore, increased serum Cr levels are associated with improved survival, whereas lower serum Cr levels are associated with increased mortality [23]. This finding suggests that low serum Cr levels, as a marker of low muscle mass, could be related to poorer outcomes. Carrero *et al*. [24] also reported that muscle wasting measured by subjective global assessment (SGA) was associated with increased mortality. These observations suggest that muscle mass is an important predictor of mortality in CKD patients.

The relationship between UPS and muscle wasting has been demonstrated. For example, treatment with a proteasome inhibitor, *N*-benzyloxycarbonyl-Ile-Glu-(*O*-*t*-butyl)-Ala-leucinal (PSI), prevented sepsis-induced muscle atrophy in an animal model [25]. In addition, the levels of messenger RNAs encoding several 20S proteasome subunits increase under catabolic conditions despite a reduction in muscle total RNA contents [26]. Du *et al*. [27] excellently demonstrated that conditions such as hyperglycemia and uremia accelerate the degradation of myofibrillar proteins (myosin and actin) that compose 60 to 70 percent of muscle protein. These findings indicate that UPS plays a critical role in muscle wasting of catabolic patients, including CKD patients.

Recently, the presence of extracellular and circulating 20S proteasomes was identified. Zoeger *et al*. [28] reported that purified 20S proteasomes from the plasma of healthy donors and patients with autoimmune diseases were intact and functional 20S particles using morphological and biochemical techniques. Henry *et al*. [29] also confirmed that circulating 20S proteasomes contain the same subunits as intracellular proteasomes using proteomic analysis. Therefore, circulating 20S proteasomes may exert physiological functions [9].

Clinically, elevated concentrations of 20S proteasomes were demonstrated in patients with cancer of the breast, stomach, kidney, colon, lung, ovary and skin [12–14]. Jakob *et al*. [13] also reported that circulating 20S proteasome concentrations were an independent prognostic factor for survival in patients with multiple myeloma. On the other hand, significantly elevated concentrations of 20S proteasomes were found in non-cancerous patients, including burn injury, sepsis and systemic autoimmune diseases [10, 11]. Notably, these concentrations were correlated well with disease activities of autoimmune diseases [10]. Taken together, these observations suggest that circulating 20S proteasomes play a role in these pathological conditions.

One of the key findings in the present study is that the negative association of 20S proteasome levels with abdominal muscle mass remains significant even after adjusting for nPNA (Table 3). Our result indicates that the 20S proteasome level is associated with abdominal muscle mass independent of dietary protein intake, because we adjusted for nPNA as a marker of protein intake levels to clarify the role of circulating 20S proteasome in dialysis patients. Further studies are needed to clarify the effect of circulating 20S proteasomes on muscle mass of CKD patients.

The origin of circulating 20S proteasomes is not known in our cohort, but several possibilities are speculated. First, circulating 20S proteasomes may be released passively from the muscle as a result of cellular damage [30]. This passive release would allow the 20S proteasome to be used as a surrogate marker for muscle wasting in hemodialysis patients. However, the fact that its concentration appears to be independent of LDH and CPK (*rho* = 0.080, *P* = 0.494 and *rho* = 0.059, *P* = 0.614, respectively) suggests that circulating 20S proteasomes do not derive from muscle breakdown. Second, microparticles, small membrane enclosed vesicles, are a possible source of circulating 20S proteasomes [31]. Interestingly, Bochmann *et al*. [31] have reported that *in vitro* generated microparticles derived from T lymphocytes released 20S proteasomes after the incubation with sphingomyelinase. Third, exosomes, which are 50–100 nm vesicular structures, are also a potential source of circulating 20S proteasomes [32]. Recently, Lai *et al*. [32] have reported that the exosomes derived from the endosomal compartment of mesenchymal stem cells contained 20S proteasomes and might have a cardio-protective effect. Taken together, microparticles or exosomes derived from the muscle may actively release 20S proteasomes into the circulation, as reported in such particles derived from T lymphocytes or the endosomal compartment of mesenchymal stem cells, respectively. Fourth, circulating 20S proteasomes may be secreted from immuno-competent cells, including thrombocytes, dendritic cells and other antigen-presenting cells, as suggested in patients with autoimmune diseases [10]. If so, the 20S proteasomes secreted from those cells may act as immuno-proteasomes and play a role in antigen presentation, possibly for the destruction of muscle proteins [33]. Further studies are needed to clarify the origin and the role of circulating 20S proteasomes in CKD patients.

Our study has several limitations. First, a longitudinal causal relationship could not be established between the changes in plasma 20S proteasome levels and alterations in muscle mass because of the cross-sectional study design. Second, the generalizability of our conclusions is not clear because of the relatively small number of patients in our single-center cohort. The variation of plasma 20S proteasome levels is also high. Therefore, larger sample sizes and validation in different cohorts are needed to confirm our results. Third, while the 20S proteasome levels in CKD patients had a tendency to increase compared with those in healthy subjects, no statistical significance was observed. The possible reason for this statistical result is that the number in healthy subjects was small. Fourth, the muscle function such as grip strength or gait speed has not been evaluated in this study. Further studies are needed to clarify the relationship between plasma 20S proteasome levels and the muscle function.

In conclusion, plasma 20S proteasome levels are significantly and negatively associated with abdominal muscle mass in HD patients. Our findings suggest that circulating 20S proteasomes and muscle metabolism are related. Future longitudinal observations and interventional studies are warranted to establish whether this link is causal in nature.
